# Impact of Hydroxychloroquine on Mortality in Hospitalized Patients with COVID-19: Systematic Review and Meta-Analysis

**DOI:** 10.3390/pharmacy8040208

**Published:** 2020-11-05

**Authors:** Thomas S. Hong, Jimmy Gonzalez, Ronald G. Nahass, Luigi Brunetti

**Affiliations:** 1 Department of Pharmacy Practice and Administration, Ernest Mario School of Pharmacy, Rutgers, The State University of New Jersey, Piscataway, NJ 08854, USA; tsh47@scarletmail.rutgers.edu (T.S.H.); jimmy.gonzalez@pharmacy.rutgers.edu (J.G.); 2 Jersey Shore University Hospital, Neptune, NJ 07753, USA; 3 Robert Wood Johnson University Hospital Somerset, Somerville, NJ 08876, USA; rnahass@idcare.com; 4 IDCare, Hillsborough, NJ 08844, USA

**Keywords:** coronavirus disease 2019 (COVID-19), hydroxychloroquine, mortality

## Abstract

Coronavirus disease 2019 (COVID-19) caused by Severe Acute Respiratory Syndrome Coronavirus 2 (SARS-CoV-2) has caused significant health and economic havoc around the globe. One of the early agents targeted for repurposing to treat and prevent COVID-19 was hydroxychloroquine (HCQ). In this systematic review and meta-analysis, HCQ is evaluated for its potential role in decreasing mortality in hospitalized patients with COVID-19. We searched PubMed, Web of Science, and medRxiv databases using combinations of the terms “COVID-19”, “SARS-CoV-2”, “coronavirus”, “hydroxychloroquine”, and “mortality”. Articles were selected for further review based on the content of their abstracts. Studies were excluded if they were of poor methodological quality, were not based in the inpatient setting, or did not have available data to assess the primary outcome of death between patients treated with HCQ versus standard of care. Once the final dataset was compiled, a meta-analysis using the random-effects model was performed. Our search identified 14 studies involving 24,780 patients of whom 12,707 patients were on HCQ alone or in combination with other adjuvant therapies. HCQ alone or in combination with other drugs did not significantly decrease mortality in hospitalized patients with COVID-19 (odds ratio [OR], 0.95; 95% CI, 0.72–1.26; *p* = 0.732; *I*^2^ = 91.05). Similar findings were observed in all subgroup analyses. HCQ did not significantly impact mortality in hospitalized patients with COVID-19. Additional well-designed studies are essential due to the heterogeneity in available studies.

## 1. Introduction

Coronavirus disease 2019 (COVID-19) caused by Severe Acute Respiratory Syndrome Coronavirus 2 (SARS-CoV-2) has caused significant health and economic havoc around the globe. As of 25 August 2020, SARS-CoV-2 has infected more than 23.86 million individuals and caused more than 800,000 deaths worldwide [1]. Remdesivir was approved for the treatment of COVID-19 requiring hospitalization by the United States (US) Food and Drug Administration (FDA) in October 2020. Even though remdesivir has been shown to shorten the time to recovery in hospitalized patients with COVID-19 [2], remdesivir still has limited evidence supporting its mortality benefits, garnering a low-level recommendation from the National Institutes of Health (NIH) COVID-19 treatment guidelines [3]. As such, a variety of therapies (investigational and repurposed drugs) are currently under investigation for COVID-19[4].

One of the earliest agents targeted for repurposing to treat and prevent COVID-19 was hydroxychloroquine (HCQ). Support for further investigation of chloroquine and its derivative HCQ was based on in vitro data demonstrating antiviral activity and a small case series [5,6,7]. Subsequently, the case series was heavily scrutinized because of significant methodological flaws [8]. Nonetheless, widespread use of HCQ with and without azithromycin (AZI) ensued, and numerous clinical studies investigating this potential therapy were initiated. As of 25 August 2020, there were over 240 clinical studies registered on clinicaltrials.gov investigating hydroxychloroquine for COVID-19. Chloroquine was observed to increase the endosomal pH and interfere with terminal glycosylation of the angiotensin-converting enzyme 2 (ACE2) receptor in vitro, inhibiting the viral infection and spread [9,10]. HCQ’s mechanisms of action against SARS-CoV-2 have yet been clearly identified, but several studies are attempting to identify its molecular mechanism. 

The US FDA granted emergency use authorization (EUA) for HCQ and chloroquine to treat COVID-19 in certain hospitalized patients on 28 March 2020. However, due to varying sample sizes, clinical settings, adjunct therapies, and methodological challenges, many recently completed HCQ studies have led to inconclusive results. Thus, the EUA on both HCQ and chloroquine was later revoked on 15 June 2020 due to safety concerns, particularly cardiovascular toxicity [11].

As research is still divided on the impact of HCQ on mortality in patients with COVID-19, we performed a systemic review and meta-analysis of current clinical studies that tested the efficacy and safety of HCQ for hospitalized patients with COVID-19. Previous meta-analyses have been performed on the effect of HCQ on mortality, but these studies included a limited number of studies or did not filter out inpatient studies alone [12,13,14,15]. These differences are important because the severity and setting of disease may impact the effects of HCQ on mortality. Furthermore, evaluations by filtering out inpatient studies may be valuable as the use of HCQ has been studied in a variety of settings. The primary objective of this analysis was to evaluate the impact of HCQ exposure on mortality in hospitalized patients with COVID-19. Secondary analyses included the evaluation of HCQ alone, HCQ in combination with AZI, and use of an adjustment of confounding factors.

## 2. Materials and Methods

### 2.1. Search Strategy and Study Selection

We performed a systemic search of PubMed, Web of Science, and medRxiv databases until 26 August 2020 using the keywords “COVID-19”, “SARS-CoV-2”, “coronavirus”, “hydroxychloroquine”, and “mortality” to search for articles. Search strategies are provided in Appendix B (Table A4). Relevant articles were chosen for further evaluation based on a review of their titles and abstracts. Bibliographic sources of selected articles were reviewed to identify any studies not captured from our initial search strategy. The Preferred Reporting Items for Systematic Reviews and Meta-Analyses (PRISMA) framework was used to guide reporting of the systematic review and meta-analysis (Table A3) [16]. 

### 2.2. Inclusion and Exclusion Criteria

Studies were included in the analysis if patients received HCQ alone or in combination with other adjunct therapies for COVID-19 infection and were compared to a control group. Study types considered for the analysis included case-control studies, cohort studies, and clinical trials. Since the primary objective of our study was to evaluate the survival benefit of HCQ in patients with COVID-19, we excluded studies that did not report mortality. We also excluded studies that were retracted from their publishing journals. Case control and cohort studies were evaluated using the modified Newcastle Ottawa Scale (NOS) [17] and randomized controlled trials using the Jadad score [18]. Therefore, only studies deemed to be of high quality were included in the meta-analysis. 

### 2.3. Data Extraction and Study Quality

We extracted primary author name, year of publication, study design, location of the study, hospitalization status of the participants, exposure to HCQ and other adjunct therapies, and mortality from each of the selected studies. A summary of this information may be reviewed in Table 1. All data were independently assessed by at least two investigators and any discrepancies were resolved through active dialogue. Study quality was rated independently by two investigators. The modified NOS was used to assess the quality of observational studies; grade seven or above was considered the threshold for inclusion. Randomized controlled trials were evaluated using the Jadad score, and those with a score of three or above were considered to be high quality. Disagreement in grading between investigators was resolved by a third reviewer.

### 2.4. Outcomes Assessed

The main objective of the study was to assess the effect of HCQ on mortality in hospitalized patients with COVID-19. Outcomes for the analyses were extracted from studies which met our inclusion/exclusion criteria. For our primary analysis, patients who were exposed to any form of HCQ were included in the HCQ treatment group while patients who were not exposed to HCQ were included in the control group. As multiple investigational therapies, including AZI, were used to treat COVID-19 during the beginning of the pandemic, many patients in both treatment and control groups were exposed to AZI during the study. We performed subgroup analyses including mortality in HCQ alone, HCQ with in combination with AZI, and use of an adjustment on confounding factors. We applied another set of inclusion and exclusion criteria for our subgroup analyses. For the HCQ alone analysis, we excluded studies that reported greater than 20% of azithromycin use in either HCQ group or control group, unknown AZI use, or unclear AZI use. For HCQ + AZI analysis, we included studies that reported greater than 80% of AZI use in treatment group but excluded studies that reported less than 20% of AZI use in control group or studies with unclear AZI use data. A shared decision was made among the authors to use the 20% cut off to reasonably include as many studies as possible and to reflect the clinical practice during the COVID-19 pandemic. To ensure that this decision did not significantly impact our outcome, we ran an additional sensitivity analysis excluding those studies that used AZI in the control group. 

### 2.5. Statistical Analyses

As the data were extracted from a variety of study designs, populations, and geographic locations, the random-effects model was adopted for the meta-analysis. For completeness, we also evaluated the study data using the fixed-effects model. The odds ratios, 95% confidence intervals, z-values, and *p*-values were calculated using both models. Publication bias was assessed through visual inspection of the funnel plot. Heterogeneity was assessed via calculation of the *I*^2^ value. A *p*-value of less than 0.05 was considered significant for all of the inferential statistics performed. Sensitivity analyses were performed by removing outlier studies. All analyses were performed using CMA 3.0 (Comprehensive Meta-Analysis, Englewood, NJ, USA).

## 3. Results

The search strategy identified a total of 598 eligible studies from the databases. After screening the title and the abstract, 28 studies remained for in-depth review. Four studies were excluded as they did not report mortality [5,19,20,21]. Three studies were excluded as there were no reported deaths [22,23,24]. Another three studies were excluded due to the absence of control group [6,25,26]. Three studies were excluded due to poor quality based on assessment of the NOS score [27,28,29]. One study was excluded as the placebo group had potential indication bias [30]. Therefore, 14 articles were included for the final review and analysis (Table 1) [15,31,32,33,34,35,36,37,38,39,40,41,42,43]. A flow chart is presented in Appendix A (Figure A3).

These 14 studies compromised of 24780 patients of whom 12707 patients were on HCQ alone or in combination with other adjuvant therapies. All of the studies were published in 2020, and the majority of studies were observational cohort with only two randomized controlled studies. Quality of these studies was assessed using the modified NOS and Jadad score (Table A1 and Table A2). Studies represented several geographic locations including the USA, Brazil, China, France, Italy, Spain, and UK. 

### 3.1. Mortality with Any HCQ Exposure

**Figure 1:**
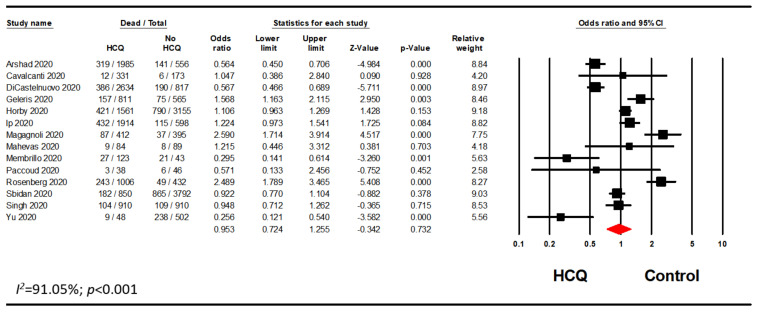
Overall meta-analysis evaluating the association between HCQ and mortality.

**Figure 2:**
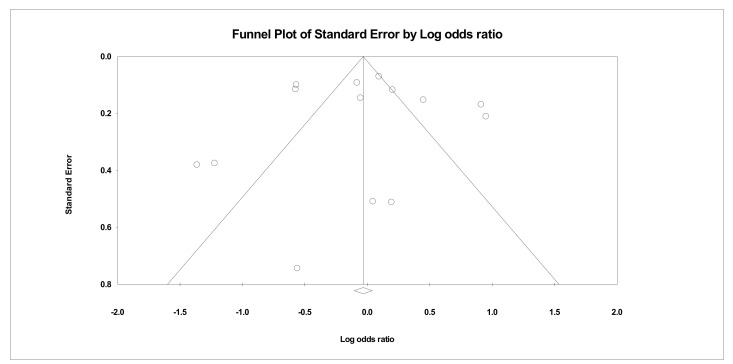
Funnel plot for overall meta-analysis.

**Figure 3:**
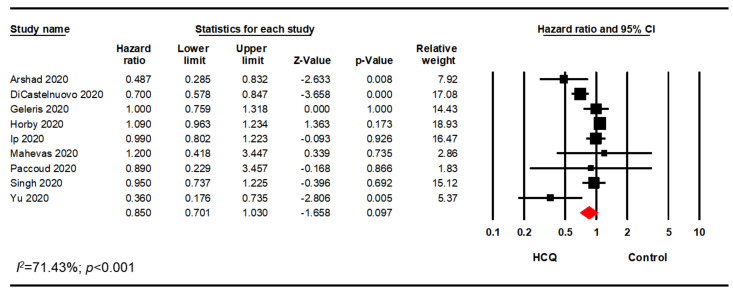
Overall meta-analysis using adjusted HR, RR, relative risk.

**Figure A1:**
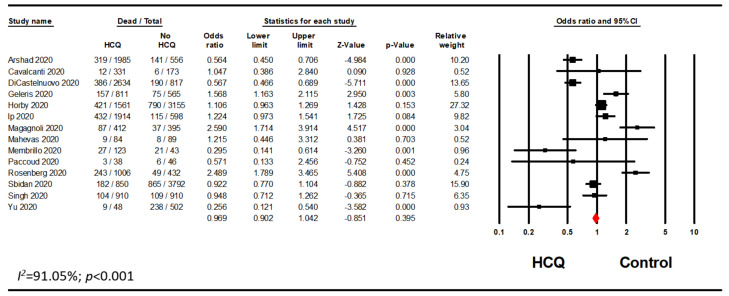
Analysis of all studies included in the meta-analysis using the fixed effects model.

A meta-analysis of all of the studies meeting inclusion exclusion criteria using raw data demonstrated that HCQ did not significantly decrease mortality of hospitalized patients with COVID-19 (odds ratio [OR], 0.95; 95% CI, 0.72–1.26; *p* = 0.732) (Figure 1). However, there was significant heterogeneity with an I^2^ value of 91.05%. As a sensitivity analysis, the analysis was rerun using a fixed effects model which did not significantly impact the mortality in hospitalized patients treated with HCQ alone or in combination with other drugs (OR, 0.97; 95% CI, 0.90–1.04; *p* = 0.395) (Figure A1). Visual inspection of the funnel plot analysis identified potential publication bias (Figure 2). The influence of the outlier studies on the overall meta-analysis was assessed by running the analysis with and without these outliers; no significant difference in outcome was identified. Furthermore, we performed the analysis using the reported adjusted hazard ratio (HR), rate ratio (RR), or relative risk from studies that adjusted mortality for potential confounders. This analysis included seven studies reporting an adjusted HR and two studies that reported either an adjusted RR or an adjusted relative risk. Meta-analysis of these nine studies aligned with the analysis of the raw data (HR, 0.85; 95% CI, 0.70–1.03; *p* = 0.097; *I*^2^ = 71.43) (Figure 3). 

### 3.2. Mortality with HCQ Alone

**Figure 4:**
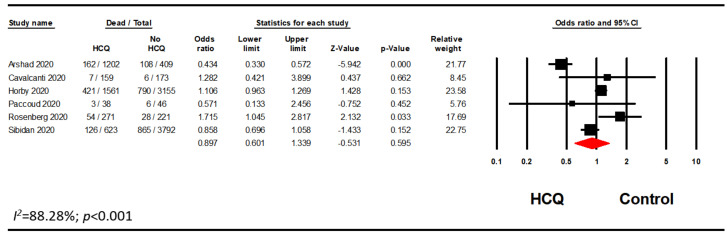
HCQ alone subgroup analysis evaluating the association between HCQ and mortality.

**Figure 5:**
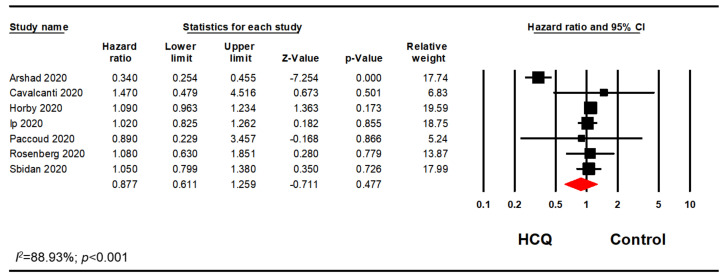
HCQ alone subgroup analysis using the adjusted HR or RR.

**Figure 6:**
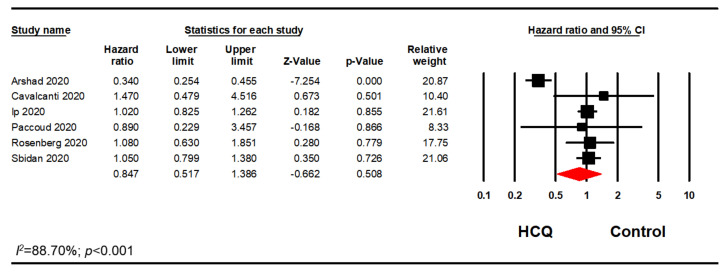
HCQ alone subgroup analysis using the adjusted HR.

**Figure A2:**
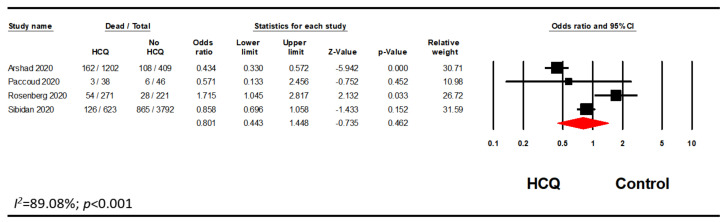
Analysis of all studies that did not use AZI in both the HCQ and the control group.

**Figure A3:**
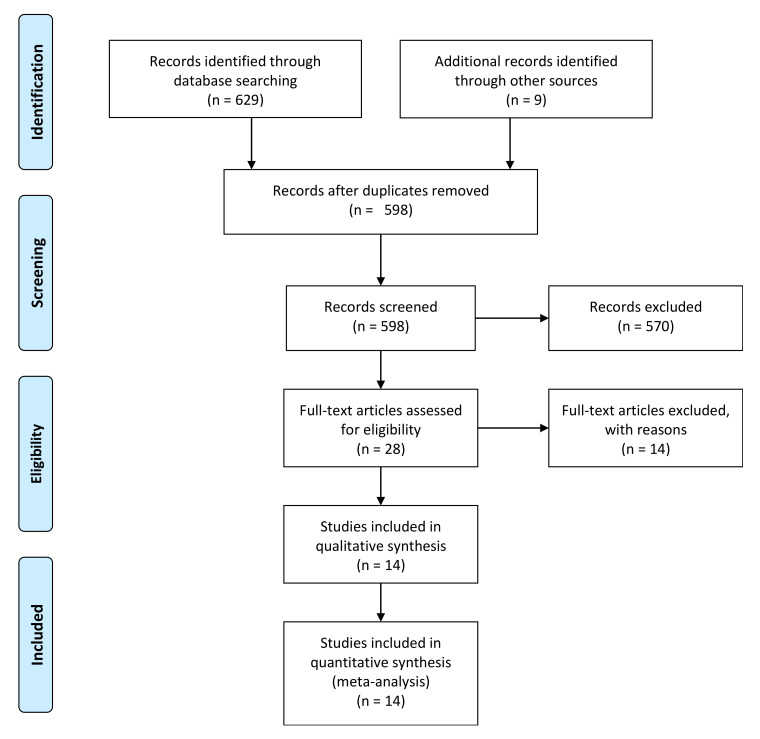
PRISMA flow diagram providing an overview of study review, selection, and inclusion in final analysis.

Many studies included patients who were on HCQ in combination with AZI, while some studies differentiated the populations by whether the patients received HCQ alone or in combination with AZI. Therefore, a subgroup analysis of patients on HCQ alone was performed. Out of 14 studies, we excluded five studies as the AZI use was greater than 20% in either treatment or control groups [15,31,34,35,37]. Two studies were excluded as there were no information on possible association with AZI [39,41]. Another one study was excluded as the data regarding AZI use were unclear [36]. This analysis included six studies and confirmed our previous finding that HCQ did not significantly reduce mortality in hospitalized patients with COVID-19 (OR, 0.90; 95% CI, 0.60–1.34; *p* = 0.595; *I*^2^ = 88.28) (Figure 4). Furthermore, a sensitivity analysis using the reported adjusted HR (n = 6) or RR (n = 1) was performed. Among those seven studies, there were no significant association between HCQ alone and mortality in hospitalized patients with COVID-19 (HR, 0.88; 95% CI, 0.61–1.26; *p* = 0.477; *I*^2^ = 88.93) (Figure 5). The results remained nonsignificant after excluding the adjusted RR from the analysis. (HR, 0.85; 95% CI, 0.52–1.39; *p* = 0.508; *I*^2^ = 88.70) (Figure 6). We ran additional sensitivity analysis excluding those studies that used AZI in the control group which included only four studies which also remained nonsignificant (Figure A2).

### 3.3. Mortality with HCQ in Combination with AZI

**Figure 7:**
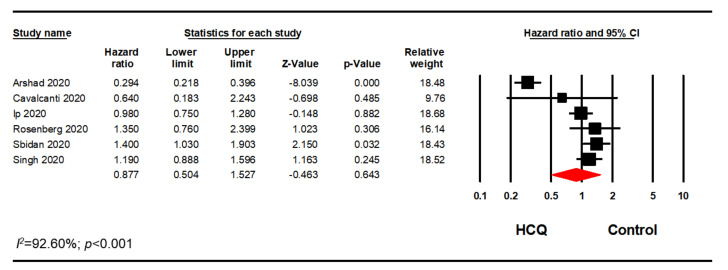
HCQ + AZI subgroup analysis evaluating the association between HCQ + AZI and mortality.

Since the studies used AZI in varying degrees, we only included studies that met our inclusion/exclusion criteria. Out of 14 studies, we excluded six studies as the AZI use was less than 80% AZI in the treatment group or greater than 20% AZI in the control group [31,34,35,37,42,43]. Another two studies were excluded as there was no information on possible association with AZI [39,41]. Therefore, a sensitivity analysis using the reported HR or relative risk on six studies was performed. This analysis included five studies that reported adjusted HR and one study that reported adjusted relative risk. Meta-analysis of these six studies confirmed our previous finding that HCQ did not significantly reduce mortality in hospitalized patients with COVID-19 (HR, 0.88; 95% CI, 0.50–1.53; *p* = 0.643; *I*^2^ = 92.60) (Figure 7).

## 4. Discussion

The outbreak of SARS-CoV-2 and COVID-19 has spread globally to become a pandemic of extraordinary proportions, drawing comparison to the past epidemic of SARS and pandemic of the 1918 “Spanish flu” [44]. Little headway has been made in the identification and development of effective treatments for afflicted patients. Guidelines issued by the WHO and US National Institutes of Health (NIH) offer recommendations for a handful of effective treatments (e.g., remdesivir, corticosteroids); however, great time and effort has been dedicated to vetting the clinical effectiveness and safety of in vitro studies or anecdotal evidence supporting vitamin therapies, HCQ, antibiotics, and other antiviral agents [3]. Consolidation of the best available evidence within a systematic review and meta-analysis provides greater certainty on the usefulness of treatment modalities with equivocal or varying benefit claims. 

In this meta-analysis, 12 observational studies and two randomized controlled studies were evaluated to assess the effect of HCQ on the mortality of hospitalized patients with COVID-19. Since we did not specify disease severity in our inclusion/exclusion criteria, patients included in this analysis presented with wide ranging clinical symptoms. Our analysis demonstrated HCQ use in hospitalized patients with COVID-19 did not significantly decrease mortality, despite both HCQ alone and HCQ in combination with AZI generating reduced point estimates of mortality in hospitalized patients with COVID-19. 

Strengths of this analysis include use of two author-independent review of all identified studies using the Newcastle-Ottowa and Jadad scales to validate study strength for inclusion in meta-analysis. Furthermore, we removed papers that were retracted from their publishing journal from our final analysis. This analysis takes into account many important variables including the hospitalization status, study design, and use of HCQ with or without AZI. Limitations of this study include variability in study design, lack of a set definition for mortality, and use of inconsistent data between the preprints and the publications. Inclusion of patients exposed to other adjuvant therapies such as AZI and other antibiotics in the control group may complicate assessment of the true impact of HCQ on mortality. Lastly, as most of the studies included in our analyses were observational studies, different types of bias (publication bias, selection bias) may have potentially impacted the outcome of our study. These limitations resulted in high heterogeneity for all of our analyses. 

Our meta-analysis result is consistent with another meta-analysis that used adjusted relative risk on the effects of HCQ on mortality [12] This study reported that there was no significant association between HCQ and COVID-19 mortality (RR, 0.83; 95% CI, 0.65–1.06; *I*^2^ = 83). However, the same study found that there was an increased mortality in patients who used HCQ in combination with AZI (RR, 1.27; 95% CI, 1.04–1.54; *I*^2^ = 38). This difference in findings may be due to our more stringent inclusion/exclusion criteria which only included studies that reported greater than 80% AZI use in treatment group and excluded studies that reported greater than 20% AZI use in control group. The use of AZI (and other adjuvant therapies) in the control group may have influenced the outcome. Nonetheless, in clinical practice azithromycin is commonly used on combination with a beta-lactam antibiotic for the treatment of pneumonia and many patients presenting with COVID-19 may have been treated empirically for community acquired pneumonia. Some studies that did not report azithromycin may have in fact utilized this drug in its study population. The inherent limitation of observational studies is restriction of data to what is readily available to the researcher. Moreover, we elected to be laxer on the inclusion of studies with hospitalized COVID-19 patients to increase our power. Our sensitivity analysis removing studies that included patients who received azithromycin in the control group supported our findings since the outcome did not change.

Like any medication, HCQ carries a risk of toxicity and should only be used if the benefits outweigh the risks. In particular, concern has been raised regarding the cardiovascular risk of HCQ in COVID-19 in a population with known risk for cardiac events [11]. Hypertension and cardiometabolic disease are commonly encountered in hospitalized COVID-19 patients [41,45,46]. Moreover, cardiac events were frequently seen in hospitalized patients with COVID-19 [47]. We did not evaluate cardiac toxicity in this meta-analysis due to the lack of available data on this outcome in the majority of studies. Given the absence of benefit, our results support the NIH, Infectious Diseases Society of America (IDSA), World Health Organization (WHO), American College of Physicians guidelines/recommendations that advocate the use of HCQ only within a clinical trial setting. As such there is an imbalance in the risk: benefit profile favoring increased risk.

## 5. Conclusions

HCQ alone or in combination with antibiotics was not associated with a significant reduction in mortality in hospitalized patients with COVID-19 in this systematic review and meta-analysis.

## Figures and Tables

**Table 1:** Summary of design and characteristics of studies included in the meta-analysis.

Study	Design	Location	Hospitalization	Exposure	Mortality
Arshad 2020	Observational Cohort	USA	Hospitalized	HCQ (n = 1202) vs. HCQ + AZI (n = 783) vs. AZI (n = 147) vs. Control (n = 409)	162/1202 vs. 157/783 vs. 33/147 vs. 108/409
Cavalcanti 2020	RCT	Brazil	Hospitalized	HCQ (n = 159) vs. HCQ + AZI (n = 172) vs. Control (n = 173)	5/159 vs. 3/172 vs. 5/173
Di Castelnuovo 2020	Observational Cohort	Italy	Hospitalized	HCQ (n = 2634) vs. Control (n = 817)	386/2634 vs. 190/817
Geleris 2020	Observational Cohort	USA	Hospitalized	HCQ ± AZI (n = 811) vs. No HCQ ± AZI (n = 565)	157/811 vs. 75/565
Horby 2020	RCT	UK	Hospitalized	HCQ (n = 1561) vs. Control (n = 3155)	421/1561 vs. 790/3155
Ip 2020	Observational Cohort	USA	Hospitalized	HCQ ± AZI (n = 1914) vs. Control ± AZI (n = 598)	432/1914 vs. 115/598
Magagnoli 2020	Observational Cohort	USA	Hospitalized	HCQ (n = 97) vs. HCQ + AZI (n = 113) vs. Control (n = 158)	27/97 vs. 25/113 vs. 18/158
Mahevas 2020	Observational Cohort	France	Hospitalized	HCQ (n = 84) vs. Control (n = 97)	3/84 vs. 4/97
Membrillo 2020	Observational Cohort	Spain	Hospitalized	HCQ (n = 123) vs. Control (n = 43)	27/123 vs. 21/43
Paccoud 2020	Observational Cohort	France	Hospitalized	HCQ (n = 38) vs. Control (n = 46)	3/38 vs. 6/46
Rosenberg 2020	Observational Cohort	USA	Hospitalized	HCQ (n = 271) vs. HCQ + AZI (n = 735) vs. AZI (n = 211) vs. Control (n = 221)	54/271 vs. 189/735 vs. 21/211 vs. 28/221
Sibidian 2020	Observational Cohort	France	Hospitalized	HCQ (n = 623) vs. HCQ + AZI (n = 227) vs. Control (3792)	126/623 vs. 56/227 vs. 865/3792
Singh 2020	Observational Cohort	USA	Hospitalized	HCQ (n = 1125) vs. Control (n = 2247)	104/910 vs. 109/910
Yu 2020	Observational Cohort	China	Hospitalized	HCQ (n = 48) vs. Control (n = 520)	9/48 vs. 238/520

AZI: Azithromycin, HCQ: Hydroxychloroquine, RCT: Randomized Clinical Trial.

## Appendix A

**Table A1:** Grading of observational studies included in the meta-analysis using the Newcastle Ottawa Score.

	Domain	Arshad 2020	Di Castelnuovo 2020	Geleris 2020	Ip 2020	Magagnoli 2020	Mahevas 2020	Membrillo 2020	Paccoud 2020	Rosenberg 2020	Sibidan 2020	Singh 2020	Yu 2020
Selection	Representativeness/Case Definition	1	1	1	1	1	1	1	1	1	1	1	1
	Selection of non-exposed/cases	1	1	1	1	1	1	1	1	1	1	1	1
	Ascertainment of exposure/selection of controls	1	1	1	1	1	1	1	1	1	1	1	1
	Baseline assessment/definition of controls	1	1	1	1	1	1	1	1	1	1	1	1
Comparability	Confounders identified	1	1	1	1	1	1	0	1	1	1	1	1
	Statistical adjustment	1	1	1	1	1	1	0	1	1	1	1	1
Outcome/exposure	Outcome/exposure assessment	1	1	1	1	1	1	1	1	1	1	1	0
	Follow-up/method for ascertainment of 1exposure	1	1	1	1	1	0	1	1	1	1	1	1
	A1dequacy of follow-up/non-response rate	1	1	1	1	1	1	1	1	1	1	1	1
	Total Score	7	9	9	9	9	8	7	9	9	9	9	8

**Table A2:** Grading of randomized controlled trials included in the meta-analysis using the Jadad score.

Domain	Cavalcanti 2020	Horby 2020
Randomization	2	2
Blinding	0	0
Withdrawals	1	1
Total Score	3	3

**Table A3:** PRISMA Checklist.

Section/Topic	^#^	Checklist Item	Reported on Page ^#^
**Title**			
Title	1	Identify the report as a systematic review, meta-analysis, or both.	1
**Abstract**			
Structured summary	2	Provide a structured summary including, as applicable: background; objectives; data sources; study eligibility criteria, participants, and interventions; study appraisal and synthesis methods; results; limitations; conclusions and implications of key findings; systematic review registration number.	1
**Introduction**			
Rationale	3	Describe the rationale for the review in the context of what is already known.	1–2
Objectives	4	Provide an explicit statement of questions being addressed with reference to participants, interventions, comparisons, outcomes, and study design (PICOS).	2
**Methods**			
Protocol and registration	5	Indicate if a review protocol exists, if and where it can be accessed (e.g., Web address), and, if available, provide registration information including registration number.	Available upon request
Eligibility criteria	6	Specify study characteristics (e.g., PICOS, length of follow-up) and report characteristics (e.g., years considered, language, publication status) used as criteria for eligibility, giving rationale.	2–3
Information sources	7	Describe all information sources (e.g., databases with dates of coverage, contact with study authors to identify additional studies) in the search and date last searched.	2
Search	8	Present full electronic search strategy for at least one database, including any limits used, such that it could be repeated.	2, Table A4
Study selection	9	State the process for selecting studies (i.e., screening, eligibility, included in systematic review, and, if applicable, included in the meta-analysis).	2–3
Data collection process	10	Describe method of data extraction from reports (e.g., piloted forms, independently, in duplicate) and any processes for obtaining and confirming data from investigators.	3
Data items	11	List and define all variables for which data were sought (e.g., PICOS, funding sources) and any assumptions and simplifications made.	3
Risk of bias in individual studies	12	Describe methods used for assessing risk of bias of individual studies (including specification of whether this was done at the study or outcome level), and how this information is to be used in any data synthesis.	4
Summary measures	13	State the principal summary measures (e.g., risk ratio, difference in means).	4
Synthesis of results	14	Describe the methods of handling data and combining results of studies, if done, including measures of consistency (e.g., I^2^) for each meta-analysis.	4
Risk of bias across studies	15	Specify any assessment of risk of bias that may affect the cumulative evidence (e.g., publication bias, selective reporting within studies).	4
Additional analyses	16	Describe methods of additional analyses (e.g., sensitivity or subgroup analyses, meta-regression), if done, indicating which were pre-specified.	4
**Results**			
Study selection	17	Give numbers of studies screened, assessed for eligibility, and included in the review, with reasons for exclusions at each stage, ideally with a flow diagram.	4–5, Figure A3
Study characteristics	18	For each study, present characteristics for which data were extracted (e.g., study size, PICOS, follow-up period) and provide the citations.	4–5, Table 1
Risk of bias within studies	19	Present data on risk of bias of each study and, if available, any outcome level assessment (see item 12).	5, Figure 2
Results of individual studies	20	For all outcomes considered (benefits or harms), present, for each study: (a) simple summary data for each intervention group (b) effect estimates and confidence intervals, ideally with a forest plot.	5–7, Figure 1 and Figure 3
Synthesis of results	21	Present results of each meta-analysis done, including confidence intervals and measures of consistency.	5–7, Figure 1 and Figure 3
Risk of bias across studies	22	Present results of any assessment of risk of bias across studies (see Item 15).	5, Figure 2
Additional analysis	23	Give results of additional analyses, if done (e.g., sensitivity or subgroup analyses, meta-regression [see Item 16]).	Figure 4, Figure 5, Figure 6, Figure 7, Figure A1 and Figure A2
**Discussion**			
Summary of evidence	24	Summarize the main findings including the strength of evidence for each main outcome; consider their relevance to key groups (e.g., healthcare providers, users, and policy makers).	8–9
Limitations	25	Discuss limitations at study and outcome level (e.g., risk of bias), and at review-level (e.g., incomplete retrieval of identified research, reporting bias).	8
Conclusions	26	Provide a general interpretation of the results in the context of other evidence, and implications for future research.	8–9
**Funding**			
Funding	27	Describe sources of funding for the systematic review and other support (e.g., supply of data); role of funders for the systematic review.	9

**^#^** Short for page number.

## Appendix B

**Table A4:** Full electronic search strategy.

**PubMed**	https://pubmed.ncbi.nlm.nih.gov/?term=%28%28Hydroxychloroquine%29+AND+%28COVID-19+or+SARS-CoV-2+or+Coronavirus%29%29+AND+%28Mortality%29&sort=date&filter=datesearch.y_1&filter=hum_ani.humans (Hydroxychloroquine) AND (COVID-19 OR SARS-CoV-2 OR Coronavirus) AND (Mortality)
**MedRxiv**	https://www.medrxiv.org/search/Hydroxychloroquine%252BCOVID-19%252BMortality%252B Hydroxychloroquine COVID-19 Mortality
